# Polymeric nanoparticles containing kojic acid induce structural alterations and apoptosis-like death in *Leishmania* (*Leishmania*) *amazonensis*

**DOI:** 10.3389/fphar.2024.1331240

**Published:** 2024-01-23

**Authors:** Poliana Queiroz-Souza, Adan Galue-Parra, Lienne Silveira Moraes, Caroline Gomes Macedo, Ana Paula Drummond Rodrigues, Victor H. S. Marinho, Fabricio H. Holanda, Irlon M. Ferreira, Edilene Oliveira da Silva

**Affiliations:** ^1^ Laboratory of Structural Biology, Institute of Biological Sciences, Federal University of Para, Belém, Pará, Brazil; ^2^ National Institute of Science and Technology in Structural Biology and Bioimaging, Rio de Janeiro, Brazil; ^3^ Laboratory of Electron Microscopy, Department of Health Surveillance, Ministry of Health, Evandro Chagas Institute, Belém, Pará, Brazil; ^4^ Laboratory of Biocatalysis and Applied Organic Synthesis, Federal University of Amapá, Macapá, Amapá, Brazil

**Keywords:** leishmanicidal activity, nanoparticle, kojic acid, silk fibroin, drug delivery system

## Abstract

Leishmaniasis encompasses a cluster of neglected tropical diseases triggered by kinetoplastid phatogens belonging to the genus Leishmania. Current therapeutic approaches are toxic, expensive, and require long-term treatment. Nanoparticles are emerging as a new alternative for the treatment of neglected tropical diseases. Silk Fibroin is a biocompatible and amphiphilic protein that can be used for formulating nanoemulsions, while kojic acid is a secondary metabolite with antileishmanial actions. Thus, this study evaluated the efficacy of a nanoemulsion, formulated with silk fibroin as the surfactant and containing kojic acid (NanoFKA), against promastigote and amastigote forms of *Leishmania* (*Leishmania*) *amazonensis*. The NanoFKA had an average particle size of 176 nm, Polydispersity Index (PDI) of 0.370, and a Zeta Potential of −32.3 mV. It presented inhibitory concentration (IC_50_) values of >56 μg/mL and >7 μg/mL for the promastigote and amastigote forms, respectively. Ultrastructural analysis, cell cycle distribution and phosphatidylserine exposure showed that NanoFKA treatment induces apoptosis-like cell death and cell cycle arrest in *L.* (*L.*) *amazonensis*. In addition, NanoFKA exhibited no cytotoxicity against macrophages. Given these results, NanoFKA present leishmanicidal activity against *L.* (*L.*) *amazonensis*.

## Introduction

The World Health Organization estimates that each year, approximately 900.000 to 1 million new cases of leishmaniasis emerge, impacting communities deprived of essential sanitation and housing, and grappling with malnutrition (OPAS, 2023; WHO, 2023). Thus, leishmaniasis is considered to be a neglected disease, as it primarily affects low-income populations and receives limited research funding to improve our comprehension of the disease, produce effective drugs and achieve disease control (Luna and Campos, 2020; Cantanhêde et al., 2021). Although leishmaniasis disease is an ancient disease with a significant epidemiological impact, current treatment is associated with several disadvantages of chemotherapy, such as adverse side effects, high cost and parasitic resistance (Akabari et al., 2021). Pentavalent antimonials are available as the first line of protocol therapy, followed by amphotericin B; however, these medications are associated with limiting factors such as: cardiotoxicity, nephrotoxicity and the resistance of some strains of the parasite. Currently, miltefosine is also used as an oral therapy; however, its use is associated with teratogenic factors (Pradhan et al., 2022).

Due to the complexity of the disease and its highly toxic treatment, the search continues for alternative therapies that can offer treatments with low toxicity and greater effectiveness (Alviano et al., 2022). In the pursuit of new bioproducts with activity against leishmaniasis, our research group has shown the potential of Kojic Acid (KA), a secondary metabolite produced by fungi, as an anti-leishmanial agent, inducer of macrophage activation and immunomodulator (Rodrigues et al., 2011; Rodrigues et al., 2014; Da Costa et al., 2018). Although KA is considered to be an effective active leishmanicidal agent, it is unstable and sensitive to light and heat (Gallarate et al., 2004; Ephrem et al., 2017). Thus, strategies that can improve the stability of this compound, such as the use of nanoparticles, are of interest (Gallarate et al., 2004). In this regard, Silk fibroin (SF), a protein synthesized by silkworms, has been extensively studied in the biomedical industry as a biomaterial for controlled drug delivery systems. The versatile properties of SF enable its use for new biomedical applications (Nguyen et al., 2019; Lujerdean et al., 2022) due to its biocompatibility with different types of cells (Yucel et al., 2014; Lujerdean et al., 2022). As such, SF represents an excellent biomaterial for the formulation of nanoparticles that increase the biological effectiveness of diverse substances, owing to its biocompatibility and mechanical properties (Rockwood et al., 2011; Pires and De Moura, 2017; Sarquis et al., 2020). Nanoparticles are carrier systems for drug delivery, with the potential to improve the bioavailability of active agents (Araujo, 2020; Holanda et al., 2023b). They are able to increase the stability of these agents in aqueous medium and, when formulated with hydrophobic polymers, can improve their capacity to destroy intracellular pathogens (Sarwar et al., 2017; De Moraes et al., 2018). Moreover, KA has previously demonstrated greater chemical and physical stability in its emulsion form (Sato et al., 2007). Thus, this study aimed to test the efficacy of the nanoparticles formulated with SF containing Kojic acid (NanoFKA), as there are currently no reports about this formulation as a leishmanicidal agent in the literature. Therefore, we herein provide information regarding the mechanisms which this formulation exerts activity against *L*. (*L*.) *amazonensis*.

## Materials and methods

### Preparation of the silk fibroin solution

The silk fibroin solution was prepared based on the method developed by Ferreira et al., 2017. Silkworm cocoons (3.0 g, from Bratac, Brazil) were degummed in a boiling (2%) (w/v) Na_2_CO_3_ solution for 30 min. The resultant fibers were filtered and washed with distilled water (3 × 500 mL). Subsequently, silk fibers were dissolved in a ternary solution (50 mL) of H_2_O:EtOH:CaCl_2_ (8:2:1 M proportions) at 30°C for 4 h. This mixture was then dialyzed (cellulose tube with an exclusion limit of 16 kDa, from Viskase, Brazil) for 3 days at room temperature, and the water was exchanged every 24 h. The fibroin solution was centrifuged (4.000 rpm for 10 min) to remove impurities and larger particles. The concentration of the silk fibroin solution was adjusted to 2% (w/w).

### Preparation and characterization of fibroin nanoparticles with kojic acid

The nanoparticles were prepared based on the method described by Marinho et al., 2022 with slight modifications. The nanoparticles were produced by an emulsification process, using water with 6% kojic acid and 2% silk fibroin solution. Initially, the KA and a mixture of ethanol and isopropanol (1:1) were mixed under constant magnetic agitation (300 rpm) for 5 min. The aqueous phase containing a silk fibroin solution was then slowly added, and the system was continuously agitated for 30 min using a vortex. Nanoparticles were stored at refrigerator temperature (4°C) after preparation. The droplet size, PDI and zeta potential of the nanoparticles were determined using a ZS zetasizer (Malvern, United Kingdom). Each sample was diluted with distilled water (1:10) to avoid multiple scattering effects, in accordance with Sarquis et al., 2020, and all measurements were made in triplicate at 25°C. The average droplet size was expressed as mean diameter.

### Structural analysis of nanoparticles

Ultrastructural analysis of the NanoFKA produced was carried out using aTransmission Electron Microscopy (TEM) JEM 400-FS microscope (JEOL Ltd., Tokyo, Japan) operated at 80 kV. For sample preparation, a drop of the suspension was deposited on formvar/carbon-coated copper grids 300 mesh (Electron Microscopy Sciences, Holfield, PA, United States) (Gélvez et al., 2021). After 60 s, the excess was gently dried with filter paper and the grid was stained using a drop of 2% w/v of uranyl acetate solution (Sigma Aldrich, St. Louis, MO, United States) for 120 s. The staining solution was gently eliminated with filter paper and the grid was rapidly dipped in particle-free ultrapure water to further eliminate loosely bound material and excess staining.

### Fluorescence analysis

The fluorescence spectra of the aqueous silk solution (NanoF, 1.0 mg/mL) and NanoFAK (1.0 mg/mL) were recorded with a HITACHI F-7000 Fluorescence Spectrophotomer, with an excitation wavelength of 310 nm. All experiment were carried out at room temperature (25°C ± 1°C) (Li et al., 2021). Data collection was accomplished using the OriginPro^®^ 8 program and the baseline measurement was performed automatically by the spectrometer.

### Animal and ethical statements

Male BALB/c albino mice (*Mus musculus* species) 6–8 weeks of age, were used in this study. The experimental protocol used was approved by the Committee on the Ethics of Animal Experiments (no 1783301019-ID 001295) of the Federal University of Para, and the experiments were carried out in accordance with the Brazilian animal protection law (law 11794/08) and in compliance with the National Council for the Control of Animal Experimentation (CONCEA, Brazil).

### Cytotoxicity assay

Macrophages were harvested from the peritoneal cavities of male BALB/c mice into Dulbecco’s modified Eagle’s medium (DMEM). Cells were incubated at 37°C in an atmosphere containing 5% CO_2_ for 1 h. Non-adherent cells were washed away with phosphate-buffered saline (PBS, pH 7·2), and the remaining macrophages were then maintained in DMEM supplemented with 10% FBS (Gibco^®^ Thermo Fisher Scientific) at 37°C in 5% CO_2_ for 24 h. Cell viability was quantified by the method of 3-(4,5-dimethylthiazol-2-yl)-2,5-diphenyltetrazolium bromide (MTT). Murine macrophages (1 × 10^6^ cells/mL) were cultured in 96-well plates and subjected to treatment with NanoFKA at concentrations of 5, 10, 20, 50, 100, 250, and 500 μg/mL for 72 h, at 37°C, 5% CO_2_ atmosphere. After discarding the supernatant and washing the wells with PBS, 0.5 mg/mL MTT diluted in PBS was added, and the system was incubated for 3 h under the same conditions The absorbance of the resulting solution was recorded at an optical density of 570 nm, as described (Fotakis and Timbrell, 2006). The same methodology was applied to NanoF. The CC_50_ values were determined by logarithmic regression analysis using GraphPad Prism 8.0 software.

### Parasites

*L.* (*L.*) *amazonensis* promastigotes (cepa MHOM/BR/26361) were obtained from the Evandro Chagas Institute (Ananindeua, Pará, Brazil) and maintained in Roswell Park Memorial Institute (RPMI) 1640 medium, supplemented with 10% fetal bovine serum (FBS) and 5 mM penicillin/streptomycin. Parasites were used in the logarithmic growth phase (4–5 days) for the antipromastigote assay and stationary growth phase (7 days) for the host cell parasite-interaction assay.

### Anti-promastigote assay

*L.* (*L.*) *amazonensis* promastigote*s* (10^6^ parasites/mL) were seeded in 24-well plates with RPMI and the cells were then treated with different concentrations of NanoFKA (5, 10, 20, 50, 100, 250, and 500 μg/mL) for 3 days. Promastigotes were incubated with 20 µL of MTT (2 μg/mL) for 4 h. Subsequently, 20 µL of DMSO were added to the wells, and the absorbance of the resulting solution was recorded at an optical density of 570 nm (Moraes et al., 2015). A known anti-leishmanial drug, Amphotericin B-0.5 μg/mL (AMPB was used as a positive control and NanoF (250 mg/mL). The inhibitory concentration (IC_50_) of the nanoparticles was determined by logarithmic regression analysis using GraphPad prism 8.0 software.

### Ultrastructural analysis of *L.* (*L.*) *amazonensis* promastigotes

For Scanning Eletron Microscopy (SEM), promastigotes were treated with 56 and 112 μg/mL (IC_50_ and twice IC_50_, respectively) of NanoFKA for 72 h, and were then fixed with glutaraldehyde (2.5%) in cacodylate buffer (0.1 M) for 1 h, before fixing with paraformaldehyde and glutaraldehyde (2.5%) in cacodylate buffer (0.1 M) for 1 h. The cells were post-fixed in osmium tetroxide (1%), dehydrated in graded ethanol, brought to their critical point with CO_2_, coated with gold and analyzed in a Vega Tescan III SEM (Alonso et al., 2021). For TEM analysis, promastigotes were treated and fixed as describe above and post-fixed wish osmium tetroxide (1%) and ferrocyanide (0.8%), dehydrated in graded acetone and embedded in epoxy resin. Ultrathin sections were obtained, stained with uranyl acetate/lead citrate and analyzed with an EM 900 TEM (Moraes et al., 2015).

### Annexin V-FITC–propidium iodide (PI) assay

Phosphatidylserine (PS) exposure can indicate apoptosis. Promastigotes were untreated or treated with NanoFKA (56 and 112 μg/mL) for 72 h. Next, cells were washed with PBS and stained with annexin V–FITC kit (Invitrogen) for 15 min at room temperature. Subsequently, promastigotes were incubated with 1 μg/mL of PI for 15 min. The samples were analyzed using the flow cytometer with BD FACSDiva software; 10.000 events were collected for each sample and analyzed using the Flowing Software 2.5.1 (RRID:SCR_015781). Miltefosine (40 µM for 24 h), an established inducer of apoptosis in *L.* (*L.*) amazonensis, was used as a positive control (da Silva et al., 2015).

### Cell cycle analysis by flow cytometry

Promastigotes (10^6^ parasites/mL) were treated with NanoFKA (56 and 112 μg/mL) for 72 h and then fixed with 70% methanol for 24 h. Subsequently, parasites were washed and labeled with 10 μg/mL PI and 10 μg/mL RNAse A for 45 min, in the dark. The stage of the cell cycle was determined using flow cytometry and BD FACSDiva software; 10.000 events were collected for each sample and analyzed using the Flowing Software 2.5.1 (RRID:SCR_015781). Miltefosine-treated promastigotes (40 µM), were used as a positive control.

### Anti-amastigote Assay 

Promastigotes were incubated separately with peritoneal macrophages at a host-parasite ratio of 1:10 for 3 h (at 37°C and 5% CO_2_), and the infected cells were treated with different concentrations of NanoFKA (5, 10, 20, 50, 100, 250, and 500 μg/mL) for 72 h, Glucantime (GLU-50 mg/mL) served as the positive control in the experiment. After the treatment, the cells were fixed with 4% paraformaldehyde and stained with Giemsa, and two hundred cells were counted with an Axio Scope A1Zeiss microscope and results expressed as a survival index (da Silva et al., 2015). The selectivity index (SI) was also evaluated to assess the activity of the NanoFKA against the parasite without altering macrophage viability. For this, the ratio between the macrophage CC_50_ and the IC_50_ of the antiamastigote activity was calculated (Santin et al., 2009). The IC_50_ values were determined by GraphPad Prism 8.0.

### Statistical analysis

The means ± standard deviation of at least three experiments were recorded. Differences between mean values were calculated using analysis of variance (ANOVA), followed by Dunnet or Bonferroni tests. *p* values ≤ 0.05 were considered statistically significant. IC_50_ and cytotoxicity concentration (CC_50_) values were calculated using logarithmic linear regression analysis using GraphPad Prism software (RRID:SCR_002798).

## Results

### NanoFKA characterization

**FIGURE 1:**
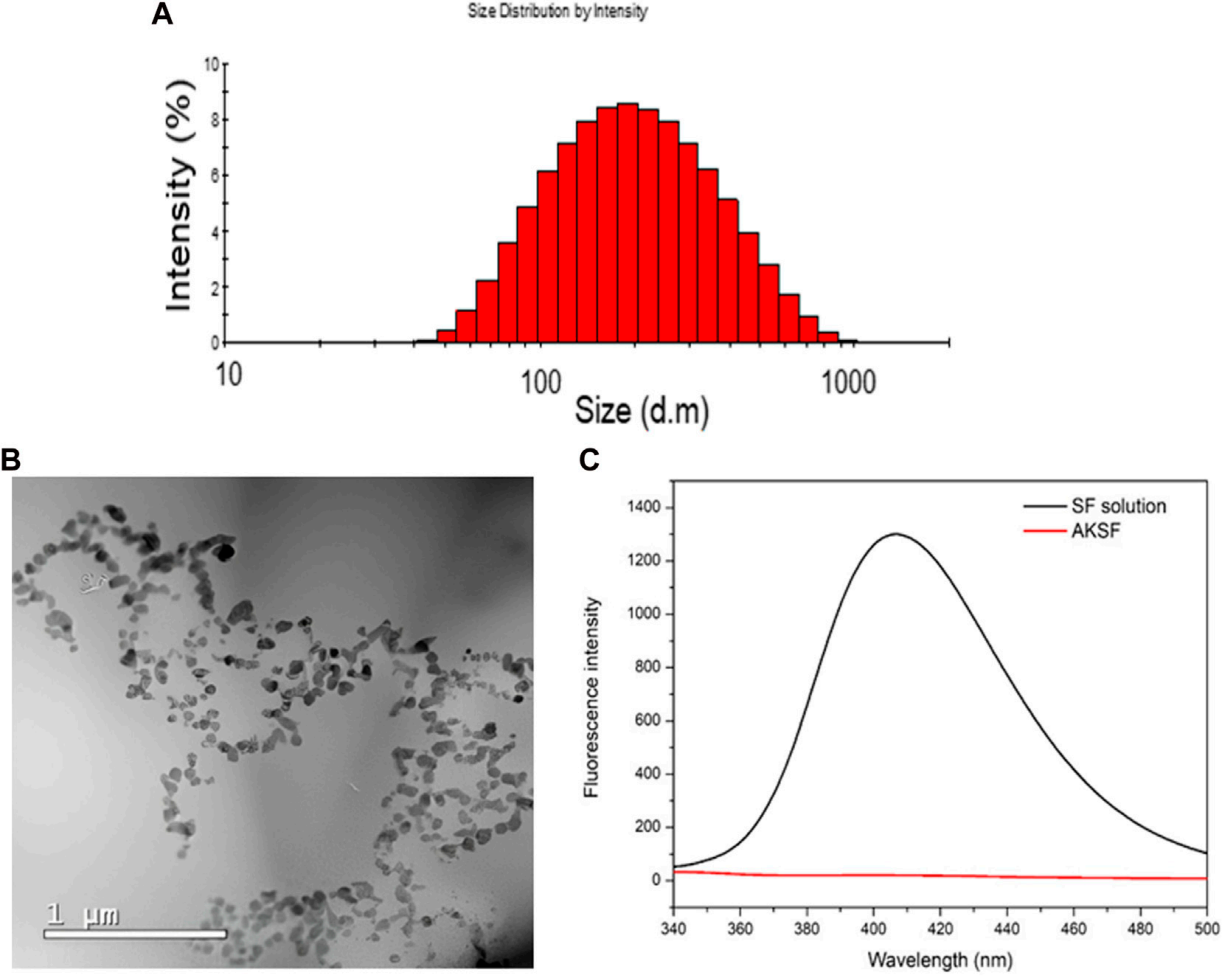
Physical chemical analysis of NanoFKA. **(A)** Dynamic Light Scattering (DLS) analysis. **(B)** TEM analysis. **(C)** Fluorescence analysis.

We successfully obtained NanoFKA with a size of 176 nm (Figure 1A), a Polydispersity Index (PDI) of 0.370, and a Zeta Potential of −32.3 mV. More negative zeta potentials are associated with greater repulsion between nanoparticles and a lower possibility of agglomeration and the zeta potential of nanoparticles can influence the interaction of the active ingredient with the cell membrane. The TEM analysis (Figure 1B) illustrates the profile of the NanoFKA nanoparticles obtained, showing spherical clusters. Figure 1C displays the fluorescence emission spectrum at 430 nm for a silk fibroin solution, compared to a solution containing the NanoFKA nanoparticle. The peak at 430 nm in the NanoF solution corresponds to tryptophan, tyrosine and cross-links (Georgakoudi et al., 2007). In contrast, the fluorescence emission profile of the silk fibroin solution containing the NanoFKA nanoparticle lacks this signal, demonstrating the interaction between the amino acid residue linkages and the KA incorporated in the lipid matrix.

### Cytotoxicity of NanoFKA against murine macrophages

**FIGURE 2:**
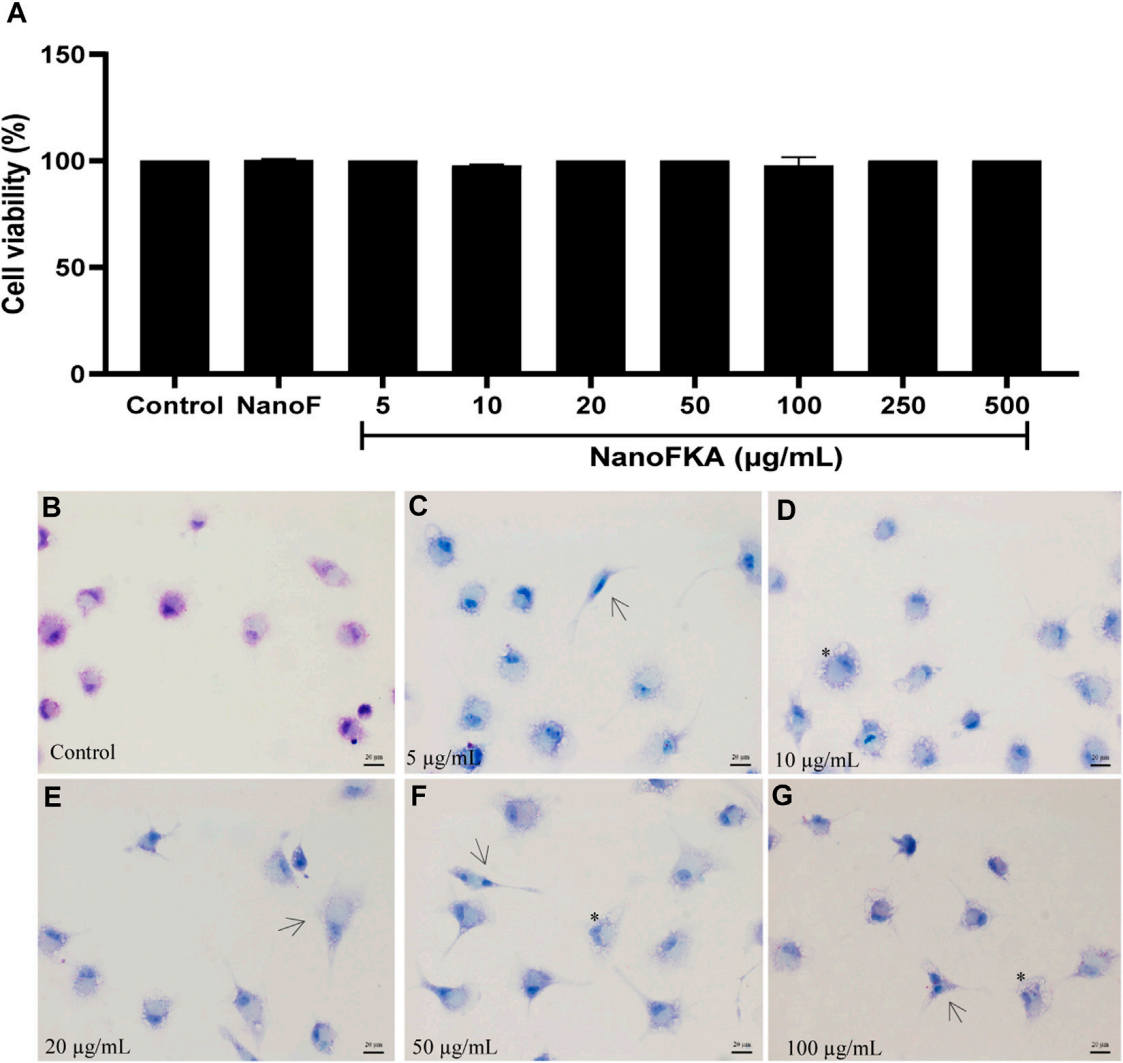
Viability of macrophages treated with NanoFKA for 72 h. **(A)** NanoFKA did not have cytotoxic effects on the cells. **(B)** Untreated macrophages (control) presented typical cell morphology. **(C–G)** Treated cells show cellular activation. NanoFKA-treated cells exhibited cytoplasmic projections (black arrow), and a large number of vacuoles (asterisks). Statistical analysis employed analysis of variance, followed by Tukey *post hoc* test *p* < 0.0001 (****), *p* < 0.001 (***), *p* < 0.01 (**), *p* < 0.05 (*).

After 72 h of incubation with NanoFKA (5, 10, 20, 50, 100, 250, and 500 μg/mL), the viability of murine macrophages was unchanged (Figure 2A) (CC_50_ > 500 μg/mL), and NanoF was not cytotoxic (CC_50_ > 500 μg/mL). A morphological analysis was carried out on peritoneal macrophages after they were incubated with different concentrations of NanoFKA. The images (Figures 2C–G) revealed that the cells treated with NanoFKA exhibited patterns of cell activation, such as spreading and the presence of vacuoles, when compared to the control group that did not receive the treatment (Figure 2B).

### NanoFKA presents leishmanicidal action against *L.* (*L.*) *amazonensis* promastigote

**FIGURE 3:**
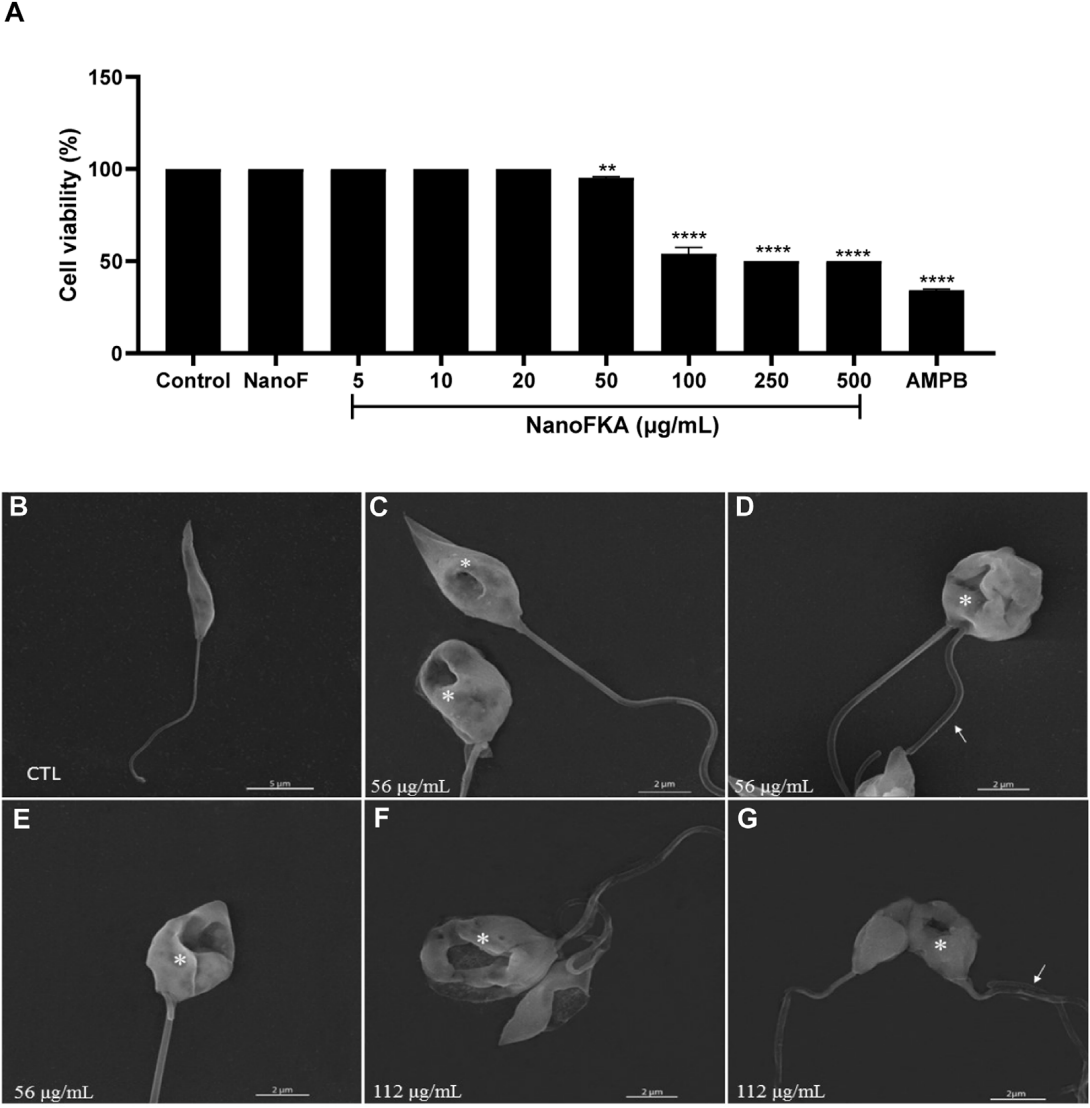
Cell viability and scanning electron microscopy analysis of the effects of NanoFKA on *L.* (*L.*) *amazonensis* promastigotes. **(A)** Promastigotes treated with 5, 10, 20, 50, 100, 250 or 500 μg/mL of NanoFKA for 72 h. Note that NanoFKA reduced the parasite number, compared to the control treatment (parasites treated with NanoF). **(B)** Control promastigote form without treatment; **(C–E)** promastigotes treated with 56 μg/mL **(F–G)** and treated with 112 μg/mL NanoFKA. Treatment reduced cell body size, modified the flagellum (white arrow), and degraded the cell body (asterisks). Statistical analysis employed analysis of variance, followed by Tukey *post hoc* test *p* < 0.0001 (****), *p* < 0.001 (***), *p* < 0.01 (**), *p* < 0.05 (*).

The *in vitro* antileishmanial activity of NanoFKA was investigated against *L.* (*L.*) *amazonensis*. After 72 h of treatment, NanoFKA demonstrated an IC_50_ of 56 μg/mL and induced a dose-dependent effect in promastigotes (Figure 3A), when compared to the untreated control. Amphotericin B (AMPB), the reference drug, was used as a positive control (IC_50_ = 0.2 μg/mL; Figure 3A), and was found to be toxic to the parasite. Control NanoF did not induce cytotoxicity in the cells. SEM analysis showed the typical morphology of untreated promastigotes (Figure 3B) and alterations and decreased cell body after treatment with NanoFKA using the IC_50_ concentration (56 μg/mL) and twice IC_50_ (112 μg/mL) (Figures 3C–G).

### NanoFKA induces ultrastructural alterations in *L.* (*L.*) *amazonensis* promastigote

**FIGURE 4:**
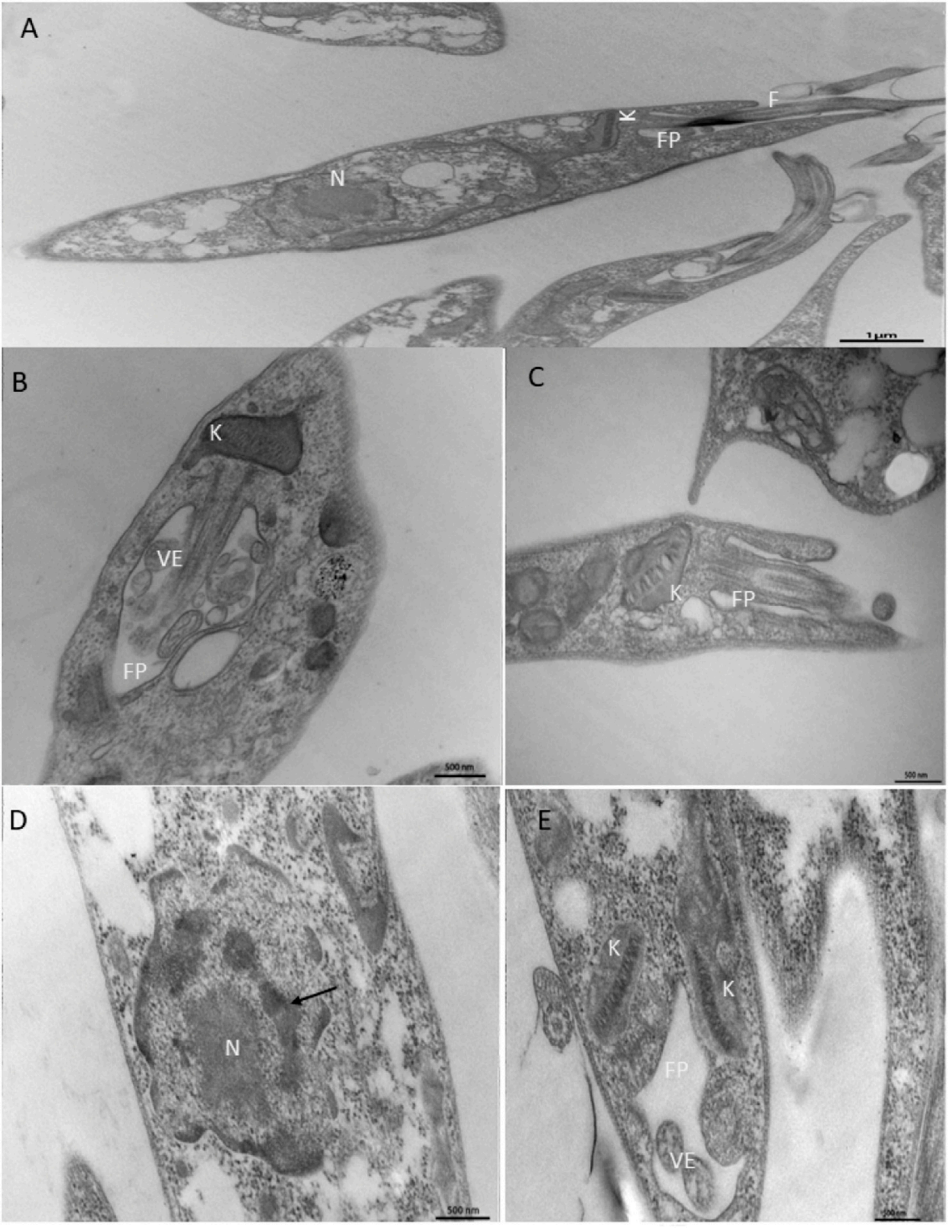
Ultrastructural analysis of the effects of NanoFKA (24 h treatment) on promastigotes of *L.* (*L.*) *amazonensis*. **(A)** Control group, untreated promastigotes with typical morphology; **(B,C)** promastigotes treated with 56 μg/mL showed accumulation of vesicles in the flagellar pocket and swelling of the kinetoplast; **(D,E)** Cells treated with 112 μg/mL. Note chromatin condensation (arrows) and vesicle accumulation inside the flagellar pocket; (K) kinetoplast; FP (flagellar pocket); N (nucleus); VE (vesicles).

TEM analysis showed that treatment of promastigotes with NanoFKA induced alterations in organelles that are essential for the survival of the parasite. In comparison to the CTL treatment (Figure 4A), which presented characteristics considered typical of promastigotes and organelles such as preserved nucleus, flagellar pocket and cytoplast, treatment with the IC_50_ concentration of NanoFKA led to swelling of the kinetoplast (Figures 4B, C). In addition, cellular disorganization was observed, as well as accumulation of vesicles in the flagellar pocket, indicating parasitic stress in response to the treatment (Figure 4B). Furthermore, parasites treated with the twice IC_50_ concentration of NANOFKA demonstrated chromatin condensation (Figure 4D), possibly indicating an apoptotic mechanism and alteration in the kinetoplast (Figure 4E).

### NanoFKA induces phosphatidylserine exposure on *L.* (*L.*) *Leishmania amazonensis* promastigote

**FIGURE 5:**
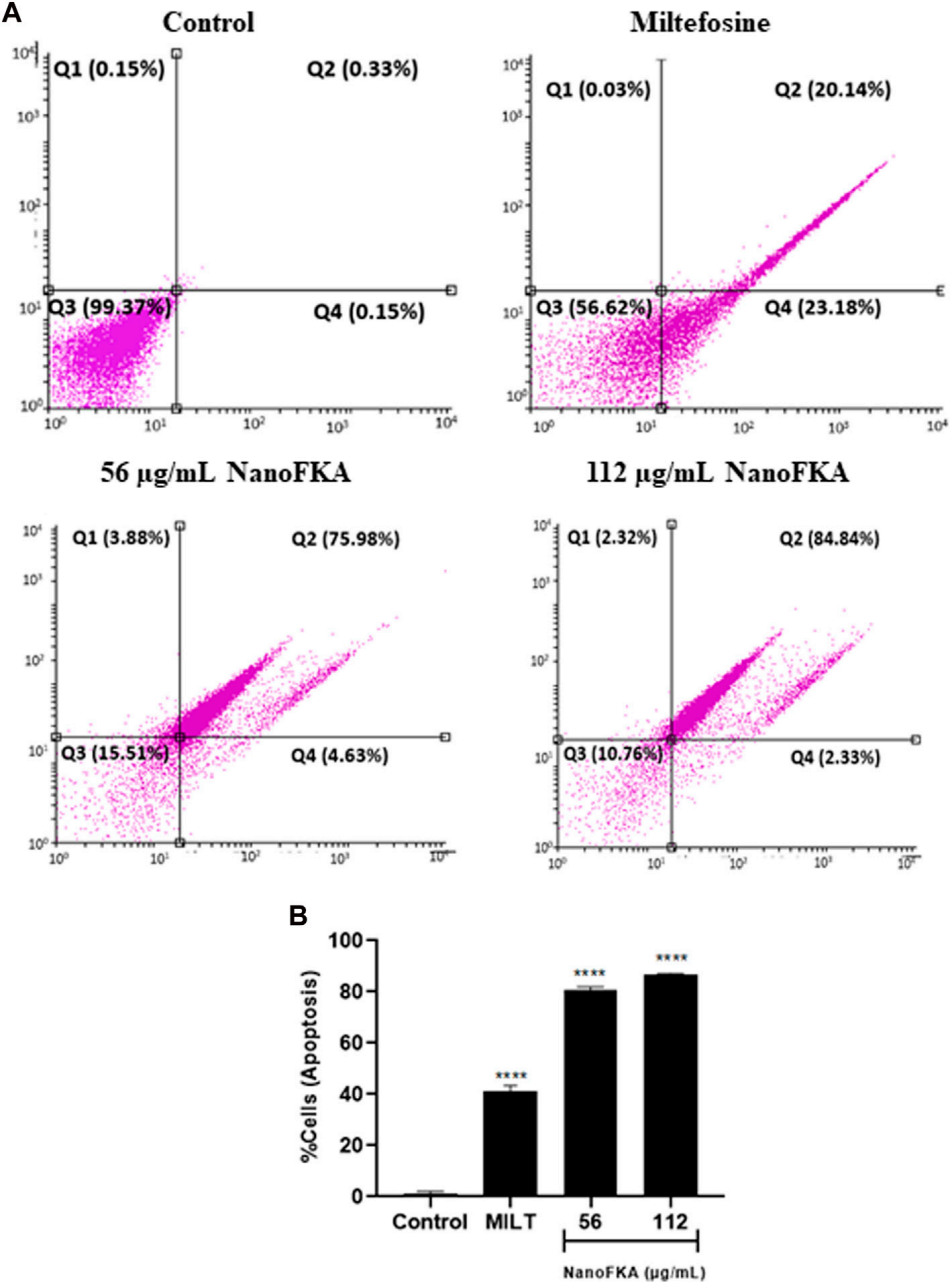
NanoFKA induces phosphatidylserine exposure on *L.* (*L.*) *amazonensis*. Cells were treated with 56 and 112 μg/mL NanoFKA, for 72 h. **(A)** Flow cytometry analysis of promastigotes double-labeled with annexin V-FITC and PI. Viable cells are in quadrant Q3, Q1 contains necrotic cells, and Q4 and Q3 contain early apoptotic cells and late apoptotic cells, respectively. **(B)** Quantification of the percentage of total apoptotic cells. Analyses were done with ANOVA and Tukey’s *post hoc* test *p* < 0.0001 (****), *p* < 0.001 (***), *p* < 0.01 (**), *p* < 0.05 (*), compared with untreated control. Positive control (40 µM miltefosine).

The annexin V–PI assay was used to confirm apoptotic like-cell death. Apoptosis of promastigotes was observed after treatment with NanoFKA at 56 μg/mL (80.61%) and 112 μg/mL (87.17%), compared to the negative control (Figure 5A, B).

### NanoFKA interfere in the cell cycle of *L.* (*L.*) *amazonensis* promastigote

**FIGURE 6:**
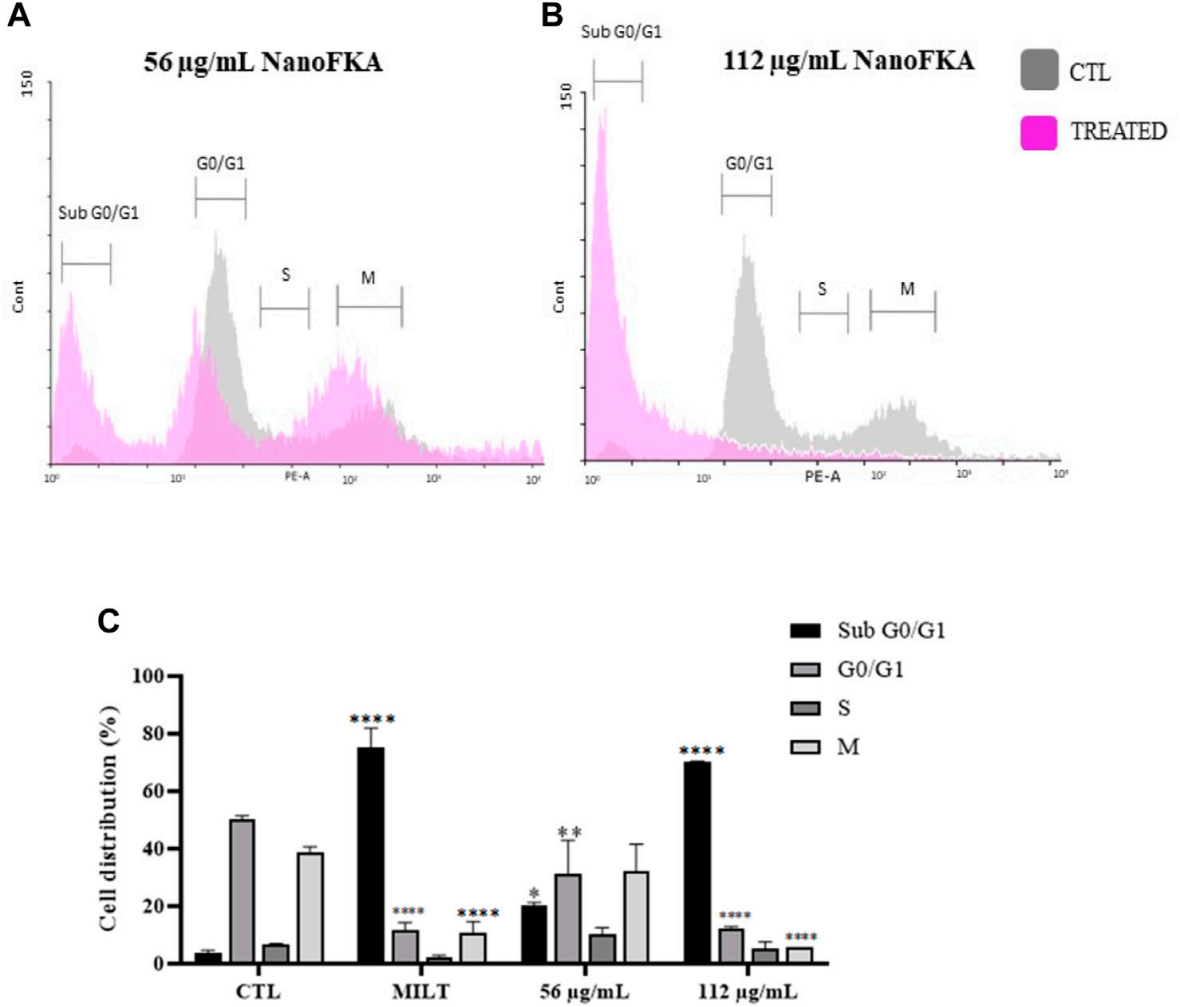
NanoFKA alters the *L.* (*L.*) *amazonensis* cell cycle. **(A,B)** The gray line indicates the negative control and the lilac line shows the parasites treated with **(A)** 56 μg/mL and **(B)** 112 μg/mL NanoFKA. **(C)** Percentage of the population in each phase of the cell cycle. The significance level was calculated using Two-Way ANOVA, followed by the Bonferroni test. *p* <0.0001 (****), *p* <0.001 (***), *p* <0.01 (**), *p* < 0.05 (*). These are representations of at least 3 independent experiments. CTL: control group. Positive control (40 µM Miltefosine).

The amount of DNA can be quantified in each of the phases of the cell cycle using specific markers, such as PI. The cell cycle of promastigotes treated with NanoFKA was analyzed by flow cytometry. After treatment, there was a significant increase in the percentage of the promastigote population in the G0 phase (16% after treatment with 56 μg/mL and 66% with 112 μg/mL NanoFKA; Figure 6), compared with the negative control. Results suggest that treated cells were undergoing apoptosis.

### Anti-amastigote assay

**FIGURE 7:**
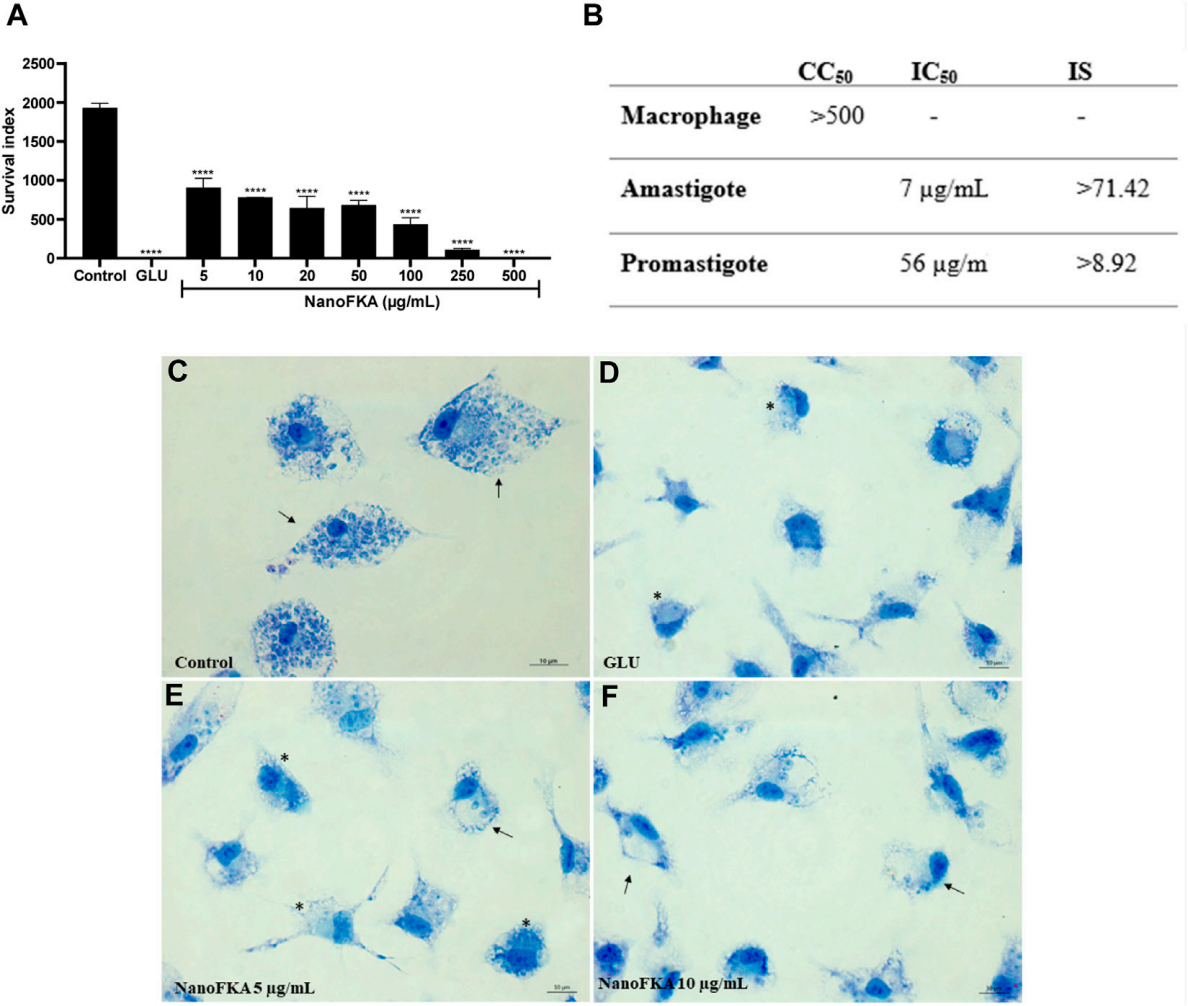
Effect of NanoFKA on amastigotes of *L.* (*L.*) *amazonensis*. **(A)** Graph showing survival index of the amastigotes; note the reduction in the number of amastigotes after the treatment with NanoFKA at different concentrations. **(B)** Table indicating IC_50_ and the survival index to amastigote and promastigote forms. **(C)** Control group without treatment; it is possible to observe several amastigotes inside the macrophages (arrows). Positive group treated with Glucantime **(D)**. Treatment with 5 μg/mL **(E)** and 10 μg/mL **(F)** NanoFKA; it is possible to observe a reduction in the number of amastigotes (arrows) and treated cells present several vacuoles without amastigotes (*). Data were obtained from 3 independent experiments. ANOVA, followed by the Tukey test. *p* < 0.0001 (****), *p* < 0.001 (***), *p* < 0.01 (**), *p* < 0.05 (*).

Incubation (72 h) of *L.* (*L*.) *amazonensis* amastigotes with NanoFKA (5, 10, 20, 50, 100, 250, and 500 μg/mL) demonstrated an IC_50_ of 7.0 μg/mL and SI of 71.42 (Figures 7A, B). Analysis by light microscopy showed that NanoFKA effectively reduces intracellular parasites (Figures 7E, F), compared with untreated infected host cells (Figure 7C). Glucantime was included as a positive control (Figure 7D).

## Discussion

In this study, we formulated a nanoparticle with KA and silk fibroin protein with the objective of improving the performance of this bioproduct. Our aim was to enhance its stability and effectiveness, thereby promoting a better leishmanicidal action against intracellular amastigotes. Nanoparticulate materials are emerging as a strategy to improve the therapeutic efficacy of molecules against neglected diseases (Sarwar et al., 2017).

Silk fibroin possesses several qualities, when compared to certain natural or synthetic polymers, such as thermal stability (Su et al., 2019), biocompatibility (Meinel et al., 2005), and increased biodistribution and bioavailability (Sarquis et al., 2020). Furthermore, the use of silk protein in biomaterial matrices enhances the effect of active substances in drug delivery processes (Holanda et al., 2023b), making it a new option for drug delivery to improve therapeutic efficiency (Chappel et al., 2008). The nanoparticle was successfully acquired and measured 176 nm in size with a PDI of 0.370, according to ISO standard documents 13321:1996 E and ISO 22412:2008. PDI values of lower than 0.7 indicate homogeneity in particle size distribution (Instruments, 2011).

Initially, we assessed whether NanoFKA would exhibit cytotoxicity towards macrophages, cells with a central role in the immune response during *Leishmania* infection. The NanoFKA did not exhibit cytotoxicity towards these cells at the different tested concentrations. These results align with the data outlined in a study conducted by Rodrigues et al. (2011), where KA did not have cytotoxic effects on peritoneal macrophages from mice after a 1-h treatment. Similar results were obtained by Da Costa et al. (2018), demonstrating that KA at different concentrations (10–100 μg/mL) also did not exhibit any cytotoxic effects on human monocytes. Furthermore, nanoparticles formulated with silk fibroin were not toxic to RAW 264.7 cells, a mouse macrophage cell line (Totten et al., 2019).

The assessment of NanoFKA’s leishmanicidal efficacy on *L*. (*L.*) *amazonensis* promastigotes proved promising, exhibiting toxicity in comparison to the control group, with an IC_50_ of 56 μg/mL. These results are in line with the observations found by Rodrigues et al. (2014), who demonstrated that KA exhibited an anti-promastigote action with an IC_50_ of 34 μg/mL. The different methodologies used in both studies may account for the variation in results obtained.

Furthermore, using the IC_50_ concentration (56 μg/mL) and twice IC_50_ concentration (112 μg/mL), we found that NanoFKA induced morphological alterations in promastigotes, including reduced cell size, flagellar changes, and perforations. These results were not well elucidated in the existing literature, whether in relation to a nanoparticle form of KA or KA alone. Therefore, additional experiments were necessary to understand what was leading to the death of this parasite.

The investigation into the mechanism of action began with TEM analyses, where significant alterations in different organelles were observed in *L.* (*L.*) *amazonensis* promastigote, when treated with NanoFKA. There was an accumulation of vesicles in the flagellar pocket, which could indicate a disruption in the parasite’s endocytosis and exocytosis processes. This phenomenon was also observed during the treatment of parasites with serine protease inhibitors, which are crucial proteins for the protozoan’s survival (Silva-Lopez et al., 2007). Furthermore, chromatin condensation was observed, a characteristic feature in apoptotic cells (Kroemer et al., 2009). These findings indicate that NanoFKA treatment may initiate apoptotic-like processes in promastigotes. Additionally, NanoFKA demonstrated the ability to induce alterations in the kinetoplast, a distinctive organelle in trypanosomatid protozoans and a potential target for drug interventions. Our findings are similar to those observed by da Silva et al. (2015) and provide important insight into the potential mechanism through which NanoFKA exerts its leishmanicidal effect, by affecting organelles and cellular processes vital for the survival of the parasite.

Other cellular events that can confirm apoptosis include alterations in mitochondrial potential, a reduction in cell size, changes in the cell cycle, and presentation of phosphatidylserine on the cell surface (Jiménez-Ruiz et al., 2010). To confirm parasite death by apoptosis mechanisms, additional tests were conducted, such as dual staining with propidium iodide (PI) and Annexin to distinguish apoptotic, necrotic, or normal cells.

NanoFKA induced late-stage apoptosis in 75.98% and 84.84% of promastigotes using the concentrations of 56 μg/mL and 112 μg/mL, respectively. These results were similar to, or higher than, those obtained for miltefosine, an apoptosis-inducing drug, which induced apoptosis in 79% of the promastigote population (Paris et al., 2004). Additionally, it is important to identify novel targets that can disrupt the parasite’s cell cycle, for its consequent elimination (Assis et al., 2021). Accordingly, NanoFKA inhibited the cell cycle of *L*. (*L.*) *amazonensis* promastigotes. This was shown by the fact that 20% and 70.18% of the protozoan population treated with the concentrations of 56 μg/mL and 112 μg/mL, respectively, were found to be in the Sub G0/G1 phase. Findings suggest that the drug causes DNA degradation, which aligns with the report of Marinho et al. (2011), regarding the positive control, miltefosine, and collectively supports the notion that the mechanism of action of NanoFKA involves the induction of apoptosis and disruption of the cell cycle of the parasite.

Finally, we attribute the effectiveness of NanoFKA to target *L.* (*L.*) *amazonensis* amastigote forms specifically. The nanoparticles demonstrated an IC_50_ of 7 μg/mL and a 62% reduction in the number of these cells inside the parasitophorous vacuole, compared to the untreated control. These findings support the initial hypothesis that nanoparticles can enhance the ability of active agents to destroy intracellular pathogens and are consistent as compared with the previous findings of our group (Rodrigues et al., 2014), which demonstrated that KA has an IC_50_ of 27 μg/mL against amastigote of *L.* (*L.*) *amazonensis*. In this current study, NanoFKA exhibited an IC_50_ of 7.0 μg/mL and a SI (selectivity index) of 71.42. According to the literature, values of greater than 10 indicate more selectivity for amastigotes than for macrophages (Oryan, 2015; Da Silva et al., 2018).

Therefore, the most important result found was against intracellular amastigotes, a replicative phase in the host macrophage and responsible for infecting other macrophages at the site of lesion. Furthermore, the mechanism of action proposed is that KA activated macrophages to kill the parasite. The combination of kojic acid with silk fibroin enhances the leishmanicidal activity of KA against intracellular forms of *L*. (*L.*) *amazonensis*, suggesting its potential use as a topical agent for the treatment of cutaneous leishmaniasis. Taken together, these results highlight NanoFKA as a promising agent for combating the intracellular forms of the parasite, and its potential application in therapeutic strategies for leishmaniasis.

## Data Availability

The original contributions presented in the study are included in the article/Supplementary material, further inquiries can be directed to the corresponding author.
